# Dulaglutide Effect on Proteins Associated With CKD Progression

**DOI:** 10.1016/j.ekir.2026.103789

**Published:** 2026-01-21

**Authors:** Brandon E. McFarlin, Sok Cin Tye, Eiichiro Satake, Zaipul I. Md Dom, Afton Kechter, Jonathan M. Wilson, Andrzej S. Krolewski, Kevin L. Duffin

**Affiliations:** 1 Diabetes, Obesity, and Complications Research Division, Eli Lilly and Company, Indianapolis, Indiana, USA; 2 Research Division, Joslin Diabetes Center, Boston, Massachusetts, USA; 3 Department of Medicine, Harvard Medical School, Boston, Massachusetts, USA

**Keywords:** biomarkers, chronic kidney disease, diabetes, dulaglutide, glucagon-like peptide-1 (GLP-1) receptor agonists

## Abstract

**Introduction:**

In the AWARD-7 clinical trial participants with type 2 diabetes mellitus (T2D) and moderate-to-severe chronic kidney disease (CKD), a once-weekly treatment with dulaglutide slowed kidney function decline compared with insulin glargine. This *post hoc* study evaluated dulaglutide’s effect on 6-month changes in plasma concentrations of 21 Joslin Kidney Panel (JKP) proteins, which were previously associated with end-stage kidney disease (ESKD) risk.

**Methods:**

Plasma concentrations of JKP proteins in participants treated with dulaglutide (*n* = 124) and insulin glargine (*n* = 125) were measured using a customized Joslin OLINK proteomic platform. Changes in circulating JKP protein concentrations from baseline to 6 months were determined.

**Results:**

Baseline JKP protein concentrations were similar between groups. After 6 months, 14 JKP proteins increased in the insulin glargine group and decreased in the dulaglutide group with statistically significant between-group differences. The most significant differences were observed for 8 tumor necrosis factor (TNF)-receptors (TNF-R1, -R2, -R3, -R4, -R6B, -R7, -R19L, and -R27), key mediators of inflammatory and apoptotic pathways. In addition, CD160, WFDC2, DLL1, LAYN, SYND1, and EPHA2 were significantly different between treatments, although to a lesser degree, and 7 other proteins remained unaffected. Kidney injury molecule 1 (KIM1), a marker of proximal tubule stress, declined in both groups without significant differences. Treatment effects were more pronounced in participants with lower baseline estimated glomerular filtration rate or higher baseline urinary albumin-to-creatinine ratio, hemoglobin A1c, or body mass index.

**Conclusion:**

Six months of dulaglutide treatment significantly lowered concentrations of 14 JKP proteins, particularly those involved in inflammatory and fibrotic pathways. These findings provide insight into biological mechanisms that may underlie the reno-protective effects of dulaglutide.

## Graphical abstract

**Figure:**
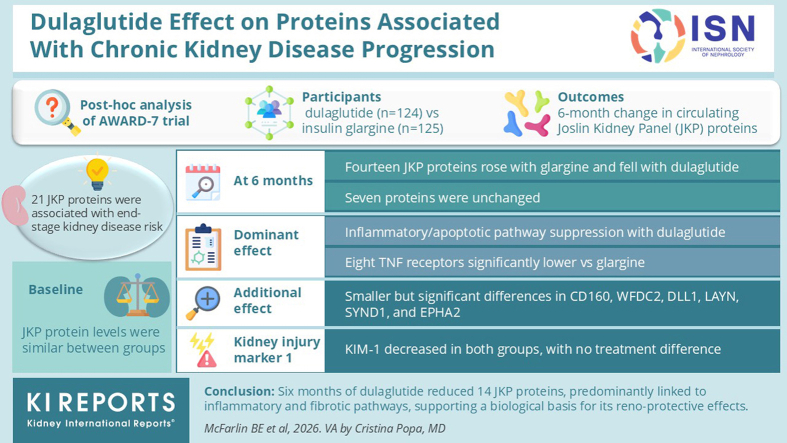

Type 2 diabetes mellitus (T2D) is a complex metabolic disorder characterized by chronic hyperglycemia, affecting over 500 million adults worldwide, and its prevalence is projected to increase in the coming decades.^1^^,^^2^ Among its diverse complications, chronic kidney disease (CKD) is estimated to affect 20% to 50% of individuals with T2D and remains a major contributor to end-stage kidney disease (ESKD) globally.^3^, ^4^, ^5^

Therapeutic advancements in diabetes pharmacotherapy, such as the use of a glucagon-like peptide-1 receptor agonists (GLP-1RAs), have shown improvement in major adverse cardiovascular events and kidney function decline in individuals with T2D.^6^^,^^7^ This class of medication not only improves glycemic control but also exhibits additional benefits, including reductions in body weight and blood pressure, as well as anti-inflammatory and anti-atherosclerotic effects.^8^^,^^9^ In the AWARD-7 trial, which evaluated the efficacy and safety of a long-acting GLP-1RA dulaglutide in participants with T2D and moderate-to-severe CKD, once-weekly dulaglutide therapy reduced the rate of kidney function decline compared with insulin glargine.^10^^,^^11^ In earlier studies within the Joslin Kidney Study cohorts, we performed large-scale proteomic analysis using 2 platforms: SomaScan (1128 circulating proteins) and OLINK (454 circulating proteins). These analyses identified 64 circulating proteins strongly associated with elevated risk of ESKD in individuals with diabetes.^12^, ^13^, ^14^, ^15^, ^16^, ^17^, ^18^ Many of these proteins were intercorrelated, reflecting activation of similar biological pathways and disease processes underlying progression to ESKD.^12^^,^^13^ To facilitate etiological research, enable screening for individuals at risk for ESKD, and support stratification for individuals based on their response to reno-protective therapies, we created a smaller panel of 21 circulating proteins, referred to as the Joslin Kidney Panel (JKP). The JKP proteins represent key circulating proteins discovered in the Joslin Kidney Study and confirmed in other studies.^19^

The 21 JKP proteins can be further categorized as follows: 9 TNF-receptors, 7 immunoregulatory or other receptors, and 5 ligands or inhibitors. Gene Ontology analysis, previous studies, and literature reviews reveal that these proteins represent TNF-receptor signaling, anti-inflammatory, profibrotic, and neuronal or axon guidance pathways.^12^, ^13^, ^14^, ^15^, ^16^, ^17^, ^18^ To quantify these 21 proteins, a custom-made Joslin OLINK proteomic platform was developed and used in the present study.^19^

This exploratory study aimed to use the Joslin OLINK proteomics platform to assess short-term (6-month) changes in the concentrations of 21 JKP proteins in participants treated with dulaglutide or insulin glargine in the AWARD-7 trial. By assessing these short-term changes, we aimed to elucidate pathways that may underlie the reno-protective benefits of dulaglutide in participants with T2D and CKD. Furthermore, we determined which of the 6-month changes in plasma concentrations of the JKP proteins could serve as predictive biomarkers to identify individuals who will benefit most from GLP-1RA therapy.

## Methods

### Treatment Groups

This *post hoc* study included a subset of participants from the AWARD-7 trial (ClinicalTrials.gov identifier: NCT01621178) who had plasma samples available at both baseline and after 6 months of treatment (*N* = 249). No additional inclusion or exclusion criteria were applied beyond the availability of plasma samples at both time points. The AWARD-7 trial was a 1-year, randomized, multicenter, open-label clinical trial that investigated the effect of once-weekly injectable dulaglutide on hemoglobin A1c (HbA1c), estimated glomerular filtration rate (eGFR), urine albumin-to-creatinine ratio (UACR), and body mass index (BMI), compared with daily insulin glargine as basal therapy.^10^ The trial included 576 participants with T2D and moderate-to-severe CKD, who were randomly assigned in a 1:1:1 ratio to receive dulaglutide 1.5 mg (*n* = 192), dulaglutide 0.75 mg (*n* = 190), or insulin glargine (*n* = 194) for 1 year.^10^

For this study, we included participants from the dulaglutide 1.5 mg (*n* = 124) and insulin glargine (*n* = 125) treatment arms who had plasma collections at baseline and after 6 months of treatment. If participants had missing time points (i.e., baseline or 6 months of treatment) they were excluded from this study. The AWARD-7 trial was conducted in accordance with the Declaration of Helsinki, the International Ethical Guidelines of Council for International Organizations of Medical Sciences, and the Good Clinical Practice Guidelines of the International Conference of Harmonization. Local institutional review boards approved the protocol at each site, and all participants provided written informed consent, which included consent to store and analyze blood samples.

### JKP Proteins and Joslin OLINK Proteomic Platform

The custom Joslin OLINK proteomic platform (Uppsala, Sweden, www.olink.com) measures plasma concentrations of 21 circulating proteins strongly associated with the risk of ESKD in individuals with diabetes.^19^ In Supplementary Table S1, we provide a list of the 21 JKP proteins and their groupings.

The Joslin OLINK platform uses Proximity Extension Assay technology to measure protein concentrations using only 20 μl of plasma or serum to measure all 21 proteins on the panel. This technology employs dual recognition by 2 matched antibodies labeled with unique DNA oligonucleotides that bind to target proteins. The platform demonstrated excellent technical performance, with each assay exhibiting high sensitivity, dynamic range, and linearity.^19^

Frozen plasma samples obtained at baseline and at 6 months of treatment with dulaglutide 1.5 mg and insulin glargine were used to measure the 21 JKP circulating proteins. The assay readouts were expressed both as Olink’s arbitrary unit (normalized protein expression) for relative quantification and as absolute concentrations in pg/ml. Quality control assessments ensured intra-assay and inter-assay coefficient variance ≤ 10% and ≤ 21%, respectively.^19^

### Statistical Analysis

All statistical analyses were conducted using R version 4.3.2 (R Project for Statistical Computing; http://www.r-project.org). Concentrations of the 21 JKP proteins (pg/ml) were log-transformed to correct for skewed distributions. Changes in concentration between baseline and 6-month treatment were expressed as a percentage change from baseline values and calculated using the formula:

$$\text{Six}-\text{month}\;\text{changes}\;\left(\%\right)\;\left(deltas\right)=\frac{\text{Concentration}\;\left(\frac{\text{pg}}{\text{mL}}\right)\text{at}\;6\;\text{month}-\text{baseline}\;\text{concentration}\;\left(\frac{\text{pg}}{\text{mL}}\right)}{\text{Baseline}\;\text{concentration}\left(\frac{\text{pg}}{\text{mL}}\right)}\;X\;100$$

Six-month changes for 4 clinical variables (i.e., eGFR, UACR, HbA1c and BMI) were calculated using a similar formula.

The effect of dulaglutide on the 6-month changes (or deltas) of clinical variables and JKP proteins was assessed by comparing the changes (or deltas) in the insulin glargine group to those in the dulaglutide group using multiple unpaired *t* tests with Welch’s correction. The same statistical test was used to analyze data stratified below and above the median values for clinical variables (i.e., eGFR, UACR, HbA1c, and BMI).

## Results

### Baseline Clinical Characteristics and Plasma Concentrations of 21 JKP Proteins According to Treatment Groups

Participants from the insulin glargine and dulaglutide 1.5 mg arm with complete plasma sample collections at baseline and 6 months were selected for this exploratory *post hoc* study. Baseline clinical characteristics were similar between the treatment groups (Table 1). The study groups were evenly balanced between men and women, with a mean ± SD age of 64 ± 8 years. The average duration of diabetes was 17.8 ± 8.5 years, and the mean HbA1c level was 8.6 ± 0.9%. The median (interquartile range) for UACR was 193 (30–653) mg/g. The average baseline eGFR, calculated using the CKD Epidemiology Collaboration-creatinine and CKD Epidemiology Collaboration-cystatin C equations, was 38 ± 13 and 39 ± 15 ml/min/1.73 m^2^, respectively. The average systolic and diastolic blood pressure was 136 ± 14 and 75 ± 9 mm Hg, respectively. Other characteristics, including body weight, BMI, serum cholesterol, and serum triglycerides, were similar between treatment groups (Table 1). Baseline plasma concentrations of the 21 JKP proteins were similar between the groups (Supplementary Table S2).

**Table 1:** Comparison of baseline clinical characteristics between individuals treated with insulin glargine and dulaglutide in the AWARD-7 trial selected for current post-hoc study

Characteristics	Insulin Glargine (*n* = 125)	Dulaglutide 1.5 mg (*n* = 124)	*P*-value
Age (yrs) mean (SD)	64.0 (8.2)	64.6 (8.5)	Ns.
Sex, Male, *n* (%)	61 (48.8)	67 (54.0)	Ns.
White, Yes, *n* (%)	89 (71.2)	89 (71.8)	Ns.
Duration of diabetes (years), mean (SD)	18.3 (8.2)	17.2 (8.7)	Ns.
Cardiovascular disease history, Yes, *n* (%)	48 (38.4)	51 (41.1)	Ns.
HbA1c (%), mean (SD)	8.5 (0.9)	8.7 (0.9)	Ns.
Systolic blood pressure (mm Hg), mean (SD)	136 (14)	136 (12)	Ns.
Diastolic blood pressure (mm Hg), mean (SD)	74.1 (8.9)	75.5 (9.1)	Ns.
HDL (mmol/l), mean (SD)	46.7 (13.3)	44.9 (13.4)	Ns.
LDL (mmol/l), mean (SD)	95.5 (32.8)	94.5 (34.5)	Ns.
TG (mmol/l), mean (SD)	185.8 (127.8)	204.8 (177.9)	Ns.
Body weight (kg), mean (SD)	89.3 (19.6)	87.2 (16.2)	Ns.
BMI (kg/m^2^, mean (SD)	32.7 (5.6)	31.9 (4.8)	Ns.
eGFR (ml/min/1.73 m^2^), mean (SD)	38.5 (12.7)	37.9 (12.9)	Ns.
eGFR ≥ 60 and < 90, *n* (%)	4 (3.2)	6 (4.8)	Ns.
eGFR ≥ 45 and < 60, *n* (%)	37 (29.6)	32 (25.8)	
eGFR ≥ 15 and < 45, *n* (%)	84 (67.2)	86 (69.4)	
UACR (mg/g), median (IQR)	187 (23–870)	199 (46–653)	Ns.
Normal albuminuria (UACR < 30), *n* (%)	36 (28.8)	21 (16.9)	0.013^a^
Microalbuminuria (UACR 30–300), *n* (%)	35 (28.0)	55 (44.4)	
Macroalbuminuria (UACR ≥ 300), *n* (%)	54 (43.2)	48 (38.7)	

BMI, body mass index; eGFR, estimated glomerular filtration rate; HbA1c, hemoglobin A1C; HDL, high-density lipoprotein cholesterol; IQR, interquartile range; LDL, low-density lipoprotein cholesterol; Ns., nonsignificant; TG, triglycerides; UACR, urinary albumin-to-creatinine ratio.

a Chi-square test for the association between UACR categories and treatment groups.

### Dulaglutide Effect on Short-Term Changes in Plasma Concentration of 21 JKP Proteins

In Table 2, we present 6-month changes (deltas) in clinical variables and plasma concentrations of the 21 JKP proteins in each treatment group separately. In participants treated with insulin glargine, values of eGFR, UACR, and HbA1c decreased, whereas BMI and plasma concentrations of most JKP proteins increased. In contrast, among those treated with dulaglutide, eGFR increased whereas the other clinical variables and most JKP proteins decreased. However, the 6-month changes (deltas) for clinical variables and the JKP proteins in each treatment group were statistically insignificant as indicated by 95% confidence interval, including negative and positive values for the deltas.

**Table 2:** Comparison of 6-month changes (%) for clinical variables and plasma concentration of 21 Joslin Kidney Panel proteins between individuals treated with insulin glargine and dulaglutide

Variables	Insulin Glargine (*n* = 125)	Dulaglutide 1.5 mg (*n* = 124)	b*P*-value
	6-mo changes^a^	6-mo changes^a^	
	(Median [IQR]), %	(Median [IQR]), %	
Clinical			
eGFR	−6 (−17.1 to 2.2)	1.1 (−10.5 to 11.9)	< 0.001
BMI	1.2 (−0.7 to 3.5)	−2.3 (−4.9 to 0.2)	< 0.001
UACR	−17 (−47.6 to 46.6)	−14.5 (−52.9 to 41.6)	Ns.
HbA1c	−11.8 (−20.8 to −4.9)	−15.4 (−21.9 to −7.2)	Ns.
TNF receptors			
TNF-R1^c^	5.5 (−5.6 to 14.1)	−1.1 (−9.5 to 5.8)	0.001
TNF-R2^c^	3.5 (−7.6 to 20.6)	−2.6 (−15.6 to 8.1)	0.003
TNF-R3^c^	3.3 (−7.5 to 17.3)	−2.9 (−13.1 to 9.9)	0.007
TNF-R4^c^	3.6 (−10.8 to 18.3)	−6.7 (−21.2 to 7.2)	< 0.001
TNF-R6B^c^	4.9 (−11.1 to 24)	−4 (−18.9 to 9.8)	< 0.001
TNF-R7^c^	1.7 (−7.8 to 19)	−2.9 (−11.5 to 8.4)	0.039
TNF-R10A	−1.8 (−11.3 to 16.3)	−1.3 (−12.5 to 11.3)	Ns.
TNF-R19L^c^	4.6 (−8.4 to 21.6)	−4.6 (−13.2 to 8.9)	< 0.001
TNF-R27^c^	5.9 (−5.8 to 23.7)	−2.1 (−11.2 to 10.7)	0.002
Immunoregulatory or other receptors			
CD160^c^	3.1 (−7.8 to 19.4)	0.7 (−12.2 to 10.4)	0.015
EPHA2^c^	5.6 (−12.9 to 25.4)	−3.4 (−19 to 17.5)	0.016
GFRa1	2.9 (−10.1 to 22.6)	−1.5 (−13.2 to 14.3)	Ns.
IL1RT1	−5.8 (−21.1 to 11.3)	1 (−15.8 to 19.8)	Ns.
KIM1	−7.8 (−21 to 13.8)	−9.2 (−27.2 to 9.5)	Ns.
LAYN^c^	3.3 (−8.8 to 20.4)	−6.1 (−17.8 to 12.5)	0.001
SYND1^c^	−0.2 (−13.5 to 17.2)	−6.7 (−18.2 to 5.5)	0.001
Ligands or inhibitors			
DLL1^c^	0.9 (−9 to 14.4)	−3.9 (−14 to 7.4)	0.012
EFNA4	2.9 (−5.7 to 16.8)	−0.1 (−8.8 to 8.9)	Ns.
PI3	2.5 (−11.8 to 22.9)	1 (−12.2 to 20.4)	Ns.
PVRL4	2.7 (−8.7 to 16.9)	0.5 (−8.9 to 12.8)	Ns.
WFDC2^c^	5.9 (−6.5 to 23.6)	−1.9 (−14.1 to 13.7)	0.004

MI, body mass index; eGFR, estimated glomerular filtration rate; HbA1c, hemoglobin A1C; IQR, interquartile range; KIM1, Kidney injury molecule 1; Ns., nonsignificant; TNF, tumor necrosis factor; UACR, urinary albumin-to-creatinine ratio.

a Six-month change in % was computed using ([6-month measurement – baseline / baseline] × 100%).

b *P*-values represent the results of the Mann-Whitney U test comparing insulin glargine versus dulaglutide.

c There were 14 key Joslin Kidney Panel proteins, for which 6-month changes were statistically significant between treatment groups. The average of combined 6-month changes for 14 relevant proteins was 3.7% ± 1.9 in insulin glargine and −3.4% ± 2.1 in dulaglutide. The difference between treatment groups was −7.1% (95% confidence interval: −8.6 to −5.6).

In Table 2, we examined differences between the deltas of the treatment groups. Regarding clinical variables, eGFR declined in participants treated with insulin glargine but increased in those treated with dulaglutide. Although BMI had the reverse pattern, BMI slightly increased during treatment with insulin glargine and decreased during treatment with dulaglutide. The differences between deltas among the treatment groups were highly statistically significant and are consistent with the original results reported for the complete clinical trial.^10^ In contrast, there was no significant difference between the treatment groups regarding the 6-month changes in UACR and HbA1c.

**Figure 1:**
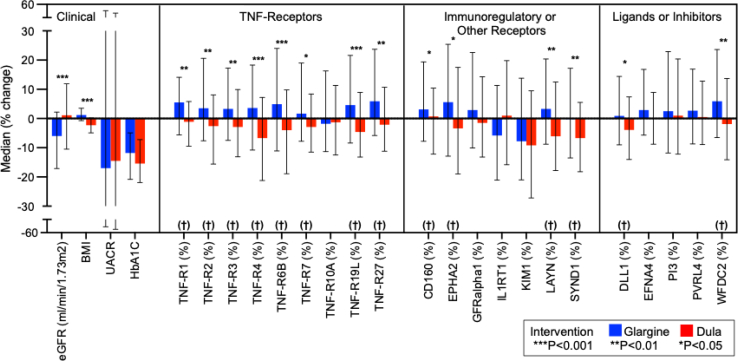
Six-month changes (%) in clinical variables and plasma concentrations of 21 Joslin Kidney Panel proteins in individuals treated with insulin glargine and dulaglutide. There were 14 key Joslin Kidney Panel proteins for which 6-month changes were statistically different between treatment groups. BMI, body mass index; eGFR, estimated glomerular filtration rate; glargine, insulin glargine; HbA1c, hemoglobin A1C; KIM1, Kidney injury molecule 1; TNF, tumor necrosis factor; UACR, urinary albumin-to-creatinine ratio.

Regarding the JKP proteins, baseline plasma concentrations of JKP proteins were similar in both treatment groups (Supplementary Table S2). After 6 months of treatment, concentrations of 14 key JKP proteins increased as measured by deltas in the insulin glargine group and decreased in the dulaglutide group. The most significant differences were observed for TNF-receptor proteins. Of the 9 TNF-receptors examined, 8 (TNF-R1, -R2, -R3, -R4, -R6B, -R7, -R19L, and -R27) increased in participants treated with insulin glargine and decreased in those treated with dulaglutide treatment; these differences were highly statistically significant. Patterns of 6-month changes for CD160, WFDC2, DLL1, LAYN, SYND1, and EPHA2 were similar to those of the TNF-receptor proteins; however, the differences were less statistically significant. Treatment with dulaglutide did not influence 6-month changes for GFRa1, IL1RT1, KIM1, EFNA4, PI3, or PVRL4. The results from Table 2 are graphically presented in Figure 1.

Altogether, 6-month changes (deltas) between treatment groups for the 14 JKP circulating proteins were statistically different. These proteins are referred to as JKP key proteins because these key proteins had similar magnitude of changes (deltas) as the result of treatment with dulaglutide.

The average combined 6-month change (delta) of the 14 key JKP proteins increased from baseline by 3.7% ± 1.9% in participants treated with insulin glargine and decreased by −3.4% ± 2.1% in those treated with dulaglutide. The average improvement in 6-month change (delta) between participants treated with insulin glargine versus dulaglutide was −7.1% ± (95% confidence interval: −8.6 to 5.6).

### Dulaglutide Effect on Short-Term Changes in Plasma Concentration of JKP Proteins According to Baseline Clinical Variables

To determine whether dulaglutide’s effect measured as 6-month changes (deltas) on circulating levels of the JKP proteins depended on clinical variables, we performed analyses stratified by baseline median values of 4 clinical variables (i.e., eGFR, UACR, HbA1c, and BMI). In Table 3 and Supplementary Figure S1, we compare 6-month changes (deltas) in circulating levels of the JKP proteins in participants treated with insulin glargine versus dulaglutide, stratified by baseline eGFR below (severe CKD) and above median (moderate CKD). In participants with severe CKD, the 6-month changes (deltas) in JKP proteins were more pronounced than those presented in Table 2 and Figure 1; deltas were more positive in participants treated with insulin glargine and more negative in those treated with dulaglutide. In contrast, in participants with baseline eGFR above median (moderate CKD), only small or no effect of dulaglutide were observed on the 6-month changes (deltas) in plasma concentration of circulating JKP proteins. When similar analyses were performed by stratifying participants with below and above median values for baseline UACR, HbA1c and BMI (Supplementary Tables S3–S5), the effect of dulaglutide on 6-month changes (deltas) in plasma concentrations of the JKP proteins showed a similar pattern to that described in Table 3.

**Table 3:** Six-month changes from baseline (%) for clinical variables and plasma concentration of 21 Joslin Kidney Panel (JKP) proteins in individuals treated with insulin glargine and dulaglutide, stratified by baseline eGFR below or above median (38 ml/min per 1.73 m^2^)

Clinical markers and JKP proteins	eGFR < median (38 ml/min per 1.73 m^2^)			eGFR ≥ median (38 ml/min per 1.73 m^2^)		
	Insulin glargine (*n* = 60)	Dulaglutide (*n* = 64)	b*P*-value	Insulin glargine (*n* = 65)	Dulaglutide (*n* = 60)	*P*-value^b^
	6-mo change^a^ median (IQR), %	6-mo change^a^ median (IQR), %		6-mo change^a^ median (IQR), %	6-mo change^a^ Median (IQR), %	
Clinical						
eGFR	−8.3 (−17.9 to 2)	5.6 (−9.1 to 17.1)	0.001	−4.9 (−16.4 to 2.4)	−1.6 (−12 to 7.3)	0.110
BMI	0.9 (−0.9 to 3.3)	−2.7 (−4.9 to −0.1)	< 0.001	1.8 (−0.6 to 3.7)	−1.7 (−4.7 to 0.8)	< 0.001
HbA1c	−12.2 (−19.8 to −2.6)	−17.2 (−23.2 to −9.8)	0.022	−11.4 (−21.1 to −6.3)	−13.2 (−20.7 to −3.2)	0.889
UACR	−15.1 (−46.5 to 40.3)	−10.4 (−47.4 to 34.9)	0.950	−19.9 (−55.8 to 61.9)	−20.1 (−58 to 77.1)	0.769
TNF receptors						
TNF- R1^c^	6.6 (−4.3 to 17.2)	−3.5 (−11 to 4.9)	0.001	3.1 (−5.6 to 11)	1.9 (−9.3 to 9)	0.265
TNF- R2^c^	5.7 (−8.6 to 24.9)	−5.1 (−18.8 to 5.6)	0.002	1.6 (−5.6 to 13.7)	0.6 (−13.9 to 13)	0.352
TNF- R3^c^	2.7 (−8 to 21.2)	−4.6 (−15.3 to 6.3)	0.013	4 (−7.2 to 15.9)	−1.3 (−9.7 to 10.1)	0.253
TNF- R4^c^	4.1 (−4.3 to 17.2)	−7.9 (−22.1 to 1.8)	< 0.001	1 (−12.2 to 18.8)	−5.3 (−21.2 to 13.4)	0.086
TNF- R6B^c^	8.2 (−11.5 to 24.4)	−4.3 (−19.5 to 3.5)	0.001	4.1 (−9.9 to 22.4)	−3.6 (−15 to 15.1)	0.089
TNF- R7^c^	3 (−7.3 to 22.7)	−3.6 (−16.2 to 4)	0.006	−0.9 (−8 to 14.9)	0.5 (−8.8 to 11.7)	0.919
TNF- R10A	−2 (−10.6 to 15.7)	−4.1 (−14.1 to 6.5)	0.142	−0.7 (−12.6 to 16.3)	3.2 (−9 to 14.9)	0.572
TNF- R19L^c^	11.4 (−4.6 to 30.1)	−5.7 (−15.3 to 8.9)	0.001	2.9 (−8.7 to 17.4)	−2.5 (−10.9 to 8.2)	0.118
TNF- R27^c^	6.9 (−8.1 to 29.1)	−4 (−14.4 to 10.6)	0.011	5 (−3 to 17.1)	−0.4 (−8.1 to 10.9)	0.074
Immunoregulatory or other receptors						
CD160^c^	2.6 (−8 to 21.9)	−3.6 (−13.9 to 10.2)	0.034	5.5 (−7.4 to 19.1)	1.1 (−9.9 to 11.2)	0.209
EPHA2^c^	8.3 (−10.9 to 23)	−5 (−18.9 to 8.8)	0.016	3.6 (−13 to 25.5)	4.9 (−19.6 to 20.8)	0.255
GFRa1	5.1 (−6.9 to 26.4)	−4.1 (−13.4 to 13.8)	0.040	−0.8 (−10.7 to 14.5)	−0.1 (−12.5 to 15.1)	0.651
IL1RT1	−3 (−20.4 to 19.3)	−0.6 (−19.6 to 19.6)	0.789	−10.1 (−22.6 to 2.8)	6.5 (−13.3 to 20.3)	0.015
KIM1	−12.1 (−26.9 to 13.5)	−5 (−28.7 to 12)	0.793	−5.5 (−17.1 to 14.1)	−10.2 (−24.5 to 3.2)	0.134
LAYN^c^	6.7 (−8.3 to 23.2)	−6.1 (−17.9 to 8.8)	0.003	2.2 (−8.8 to 16.9)	−6.5 (−17.8 to 14.5)	0.078
SYND1^c^	4.3 (−16.6 to 17.6)	−10.3 (−19.5 to 0.9)	0.002	−0.6 (−12.5 to 11.3)	−3.2 (−17.3 to 9.3)	0.173
Ligands or inhibitors						
DLL1^c^	1.9 (−7.4 to 14.9)	−4.3 (−16.4 to 5.3)	0.010	0.2 (−11.1 to 10.7)	−3.7 (−11 to 8.4)	0.439
EFNA4	4.3 (−5.4 to 19.7)	−2.1 (−9.3 to 6.9)	0.018	1.2 (−7.5 to 14.8)	1.9 (−5.7 to 9.8)	0.892
PI3	8.1 (−9.1 to 27.9)	−4.9 (−16.1 to 20.9)	0.081	−1.9 (−12.9 to 22.1)	10 (−10 to 18.3)	0.478
PVRL4	3.3 (−10.2 to 18)	0.5 (−10.8 to 10.7)	0.225	2.5 (−6.9 to 14.4)	0.6 (−7.8 to 14.1)	0.799
WFDC2^c^	8.3 (−7.2 to 26.5)	−5.3 (−14.9 to 12.2)	0.010	2.4 (−6 to 19.2)	2.3 (−12.2 to 15.3)	0.214

BMI, body mass index; eGFR, estimated glomerular filtration rate; HbA1c, hemoglobin A1C; IQR, interquartile range; KIM1, Kidney injury molecule 1; TNF, tumor necrosis factor; UACR, urinary albumin-to-creatinine ratio.

a Six-month change in % was computed using the formula: ([6-month measurement – baseline / baseline) × 100%].

b *P*-values represent the results of the Mann-Whitney U test comparing insulin glargine versus dulaglutide.

c Denotes the 14 key JKP proteins; see legend to Table 2.

A summary of the results obtained for the average 6-month changes (deltas) in concentration of the 14 key JKP proteins in stratified analyses according to medians of the 4 baseline clinical variables is shown in Table 4. Among participants with baseline eGFR below median (severe CKD) treated with insulin glargine, the average combined 6-month change (delta) for the key JKP proteins was 5.1% ± 2.8% increase from baseline; whereas among those treated with dulaglutide, the average 6-month change (delta) was −4.6% ± 1.9% decrease from baseline. Therefore, the dulaglutide effect was an average reduction in the concentration of the 14 key JKP proteins by −9.7% (95% confidence interval: −11.5% to −7.9%; *P* < 0.001).

**Table 4:** Combined 6-month changes (%) of key Joslin Kidney Panel (JKP) proteins^a^ in individuals stratified according to baseline value below and above median of each of 4 clinical variables and treatment groups

Stratification according to baseline clinical variables	Below median of clinical variables			Above median of clinical variables		
	Insulin glargine (*n* = 60)	Dulaglutide (*n* = 64)	Difference between treatment groups (Dulaglutide effect)	Insulin glargine (*n* = 65) Combined 6-month changes	Dulaglutide (*n* = 60) Combined 6-month changes	Difference between treatment groups (Dulaglutide effect)
	Averaged 6-mo changes	Averaged 6-mo changes		Averaged 6-mo changes	Averaged 6-mo changes	
	Average (SD)^b^ %	Average (SD)^b^ %	Average and (95% CI) %	Average (SD)^b^ %	Average (SD)^b^ %	Average and (95% CI) %
eGFR	5.1 (2.8)	−4.6 (1.9)	−9.7 (−11.5 to −7.9)	2.1 (2.0)	−1.0 (3.1)	−3.1 (−5.0 to −1.2)
BMI	2.4 (2.0)	0.1 (2.3)	−2.3 (−3.9 to −0.7)	3.9 (3.1)	−5.2 (3.0)	−9.1 (−11.4 to −6.8)
HbA1c	1.6 (1.8)	−1.6 (2.3)	−3.2 (−4.7 to −1.7)	4.4 (3.2)	−3.5 (3.0)	−7.9 (−10.2 to −5.6)
UACR	0.5 (1.9)	−0.7 (1.9)	−1.2 (−2.6 to 0.2)	6.4 (4.2)	−4.5 (3.2)	−10.9 (−13.7 to −8.1)

BMI, body mass index; CI, confidence interval; eGFR, estimated glomerular filtration rate; HbA1C, hemoglobin A1C, UACR, urinary albumin-to-creatinine ratio.

a 14 key JKP proteins, see legend to Table 2.

b The average and SD for combined 6-month changes (%) for relevant JKP proteins were calculated by summing up medians of each relevant JKP proteins in subgroups stratified according to baseline median values of 4 clinical variables and treatment groups. See Table 3 and Supplementary Tables S3–S5.

Among participants with baseline eGFR above median (moderate CKD), the average combined 6-month change (delta) for the 14 key JKP proteins was 2.1% ± 2.0% for those treated with insulin glargine and −1.0% ± 3.1% for those treated with dulaglutide. Therefore, the dulaglutide effect was an average reduction in the concentration of the 14 key JKP proteins by −3.1% (95% confidence interval: −5.0% to −1.2%; *P* < 0.001).

Although the effects of dulaglutide on short-term changes in concentrations of the 14 key JKP proteins were statistically significant in participants with both moderate and severe CKD, the effects were 3 times larger in those with severe CKD. Similar dulaglutide effects were observed in stratified analyses according to median UACR, HbA1c, and BMI (Table 4).

## Discussion

In this exploratory *post hoc* study in a subset of participants with T2D and moderate-to severe CKD from the AWARD-7 clinical trial, we examined the effect of a 6-month dulaglutide treatment on lowering plasma concentrations of 21 JKP proteins. These proteins represent the 64 circulating proteins, which were found previously to be strongly associated with risk of progression to ESKD in individuals with type 1 diabetes mellitus and T2D.^12^, ^13^, ^14^, ^15^, ^16^, ^17^, ^18^, ^19^ The JKP comprises 3 categories of proteins, namely: TNF receptors, immunoregulatory or other receptors, ligands or inhibitors. Of these, 6-month treatment with dulaglutide significantly reduced concentrations of 14 JKP proteins in comparison with insulin glargine treatment. Our analysis of short-term changes in JKP protein biomarkers highlights potential biological pathways, especially those involving inflammation and fibrosis, that could mediate the reno-protective effects of dulaglutide. Herein, we discuss the relevance of our findings for selecting participants for dulaglutide treatment and monitoring its effectiveness. We also explore the mechanisms through which dulaglutide may exert its reno-protective effects.

Dulaglutide, a GLP-1RA, is approved for glycemic control and reduction of major adverse cardiovascular events in adults with T2D. In addition, clinical evidence shows that dulaglutide can slow kidney function decline.^6^ However, there is a spectrum of response to GLP-1RA therapy with some individuals having more robust response than others with little response. Therefore, biomarkers that could help to identify those individuals who might benefit the most from GLP-1RA therapy and to monitor response to therapy are warranted. The AWARD-7 trial was a 1-year clinical study that provided sufficient number of outcomes to assess the beneficial effect of dulaglutide on reducing the risk of kidney-related outcomes.^10^^,^^11^ The current study, however, was unable to examine which of the JKP proteins might have been good predictors of the dulaglutide effect on reducing long-term risk of kidney outcomes because the sample size was too small and the follow-up period too short. Instead, we examined only the effect of dulaglutide on short-term changes in concentration of the 21 JKP proteins as proxies for the long-term kidney outcomes.

Combining 6-month changes (deltas) for the 14 response JKP proteins could provide a more reliable tool to assess the response to dulaglutide treatment. Monitoring the 6-month changes in concentration of the JKP protein concentrations, stratified by baseline clinical characteristics, should further discriminate those with greater therapeutic response to dulaglutide. Notably, dulaglutide was most effective in reducing plasma concentrations of JKP proteins among participants who had severe kidney function impairment (eGFR < median). This finding aligns with recent clinical studies demonstrating greater reno-protective responses with GLP-1RAs in individuals with more advanced stages (3b/4) of CKD.^20^^,^^21^ Treatment with dulaglutide was more effective in reducing circulating levels of the JKP proteins among participants with higher baseline obesity, albuminuria, or HbA1c. These novel observations suggest that the effect of dulaglutide is conditional on levels of baseline clinical variables. Thus, physicians can identify individuals who, in the long-term, may benefit the most from dulaglutide treatment regarding reduction in risk of kidney outcome by examining the 6-month decline in concentration of JKP proteins in circulation according to the baseline levels of clinical variables.

Our study provides insights into the disease processes that may underline dulaglutide’s effect on reducing risk of kidney function decline and delaying progression to kidney failure. The plasma concentrations of IL1RT1 and PVRL4 which are strong predictors of progression to kidney failure but with unknown mechanisms in the disease, were not affected by dulaglutide treatment. KIM-1, a well-known biomarker that is secreted by stressed proximal tubule cells during renal loss is linked to development of CKD.^22^^,^^23^ Elevated plasma concentrations of KIM1 may indicate early damage to proximal tubular cells and could be causally involved in this process.^24^^,^^25^ Our findings indicate that dulaglutide lowered plasma levels of KIM1 but to the same degree as insulin glargine in later stages of CKD.

In contrast, dulaglutide treatment reduced plasma concentrations of nearly all other JKP proteins, particularly TNF-receptors. Affected TNF-receptors included TNF-R1, -R2, -R4, -R6B, -R19L, and -R27, with only TNF-R10A remaining unaffected. Our previous research demonstrated that elevated levels of circulating TNF-Rs in individuals with T2D are strong predictors of progression to kidney failure.^17^^,^^26^ We recently showed that many of these TNF-receptors were upregulated in damaged proximal tubule cells from individuals with CKD and may be associated with proximal tubule cell death processes.^12^ This suggests that dulaglutide may reduce tubule cell death, leading to lower release of TNF-Rs into circulation. Alternatively, high circulating levels of TNF-Rs may indicate activation of the TNF-α pathway, contributing to insulin resistance either directly or by stimulating the inflammatory processes that lead to T2D complications.^27^ Dulaglutide treatment may alleviate this disease process, manifested as a reduction in circulating levels of TNF-R levels. This hypothesis is supported by preclinical studies showing that GLP-1RA, such as liraglutide, inhibit the TNF-α signaling pathway and protect against high-fat diet–induced insulin resistance and inflammation.^28^, ^29^, ^30^

Kidney fibrosis is a common pathological manifestation in CKD, characterized by replacement of functional tissue with excess extracellular matrix. We previously showed that dulaglutide treatment was associated with lower type VI collagen formation and higher collagen degradation than with insulin glargine.^31^ This study extends our previous findings by showing that dulaglutide treatment lowered circulating levels of profibrotic proteins such as DLL1, LAYN, and SYND1. Previous studies have shown that high circulating levels of DLL1 are good predictors of the effectiveness of fenofibrate treatment in reducing the risk of rapid kidney function decline in individuals with T2D.^15^ In addition, LAYN and SYND1 have been implicated in TNF-α–induced epithelial-mesenchymal transformation of kidney tubular cells and associated with the development of fibrosis.^32^, ^33^, ^34^ Determining whether dulaglutide influences these fibrotic pathways directly or indirectly through inflammatory or apoptotic mechanisms is beyond the scope of this study. However, it is plausible that the reno-protective benefits of dulaglutide involve the reduction of inflammation, fibrosis, or both in kidney.^35^ Importantly, the effect of dulaglutide on lowering circulating levels of key JKP proteins was most pronounced in participants with severe CKD, the stage characterized by extensive inflammation and fibrosis. In addition, dulaglutide treatment showed less increase or lowering of circulating levels of CD160, EPHA2, and WFDC2 than with insulin glargine, although these effects were less pronounced. These suggest an influence on processes beyond inflammation or fibrosis.

The strengths of this study include the use of stored samples from the AWARD-7 trial, a rigorously conducted CKD clinical trial with an active comparator arm, insulin glargine; and the use of a biomarker panel (JKP) containing validated prognostic biomarkers for progression to ESKD. However, this study is limited by its *post hoc* design and should be considered hypothesis-generating. In addition, this study did not include an independent validation cohort which limits the generalizability and results should be interpreted with that consideration. Further, at baseline, the dulaglutide group included a higher proportion of participants with microalbuminuria and fewer with normal albuminuria than the insulin glargine group; the proportion of participants with macroalbuminuria was similar between groups. This distribution suggests that, although there were differences in baseline albuminuria categories, both groups included adequate representation of participants with advanced kidney disease. Although we stratified data by baseline UACR in Supplementary Table 3, the potential impact of these differences on overall treatment outcomes should be considered when interrupting results. Lastly, the relatively small sample size, limited by plasma sample availability, necessitates replication in larger clinical trials with sufficient kidney outcomes to confirm these findings.

## Disclosure

BEM, JMW, AK, and KLD are employees and stockholders of Eli Lilly and Company. All the other authors declared no competing interests.

## Supplementary Material

Supplementary Material (PDF) **Figure S1. Six**-month changes (%) of clinical variables and plasma concentration of 21 Joslin Kidney Panel (JKP) proteins in individuals treated with glargine and dulaglutide (Dula), stratified by baseline eGFR (A) above or (B) below median of 38 ml/min per 1.73 m^2^. **Table S1.** List of 21 proteins associated with the risk of end-stage kidney disease (ESKD) included in the Joslin Kidney Panel (JKP). **Table S2.** Comparison of baseline plasma concentration (pg/ml) of 21 Joslin Kidney Panel (JKP) proteins between individuals treated with glargine and dulaglutide (Dula). **Table S3.** Six-month changes (%) for clinical variables and plasma concentration of 21 Joslin Kidney Panel (JKP) proteins in individuals treated with glargine and dulaglutide (Dula), stratified by baseline UACR below or above median (187 mg/g). **Table S4.** Comparison of 6-month changes in % for clinical variables and plasma concentration of 21 Joslin Kidney Panel (JKP) proteins between individuals treated with glargine and dulaglutide (Dula), stratified by baseline HbA1c below or above median (8.4%). **Table S5.** Comparison of 6-month changes in % for clinical variables and plasma concentration of 21 Joslin Kidney Panel (JKP) proteins between individuals treated with glargine and dulaglutide (Dula), stratified by baseline eGFR below or above median (32 kg/m^2^). CONSORT 2025 Checklist.

## Footnotes

Supplementary File (PDF)
**Figure S1. Six**-month changes (%) of clinical variables and plasma concentration of 21 Joslin Kidney Panel (JKP) proteins in individuals treated with glargine and dulaglutide (Dula), stratified by baseline eGFR (A) above or (B) below median of 38 ml/min per 1.73 m^2^. **Table S1.** List of 21 proteins associated with the risk of end-stage kidney disease (ESKD) included in the Joslin Kidney Panel (JKP). **Table S2.** Comparison of baseline plasma concentration (pg/ml) of 21 Joslin Kidney Panel (JKP) proteins between individuals treated with glargine and dulaglutide (Dula). **Table S3.** Six-month changes (%) for clinical variables and plasma concentration of 21 Joslin Kidney Panel (JKP) proteins in individuals treated with glargine and dulaglutide (Dula), stratified by baseline UACR below or above median (187 mg/g). **Table S4.** Comparison of 6-month changes in % for clinical variables and plasma concentration of 21 Joslin Kidney Panel (JKP) proteins between individuals treated with glargine and dulaglutide (Dula), stratified by baseline HbA1c below or above median (8.4%). **Table S5.** Comparison of 6-month changes in % for clinical variables and plasma concentration of 21 Joslin Kidney Panel (JKP) proteins between individuals treated with glargine and dulaglutide (Dula), stratified by baseline eGFR below or above median (32 kg/m^2^). CONSORT 2025 Checklist.
