# Supplementary material for: Dulaglutide Effect on Proteins Associated With CKD Progression
Source: Kidney Int Rep. 2026 Jan 21;11(4):103789. doi: 10.1016/j.ekir.2026.103789 (PMC12925400; doi:10.1016/j.ekir.2026.103789)
Supplement: Supplementary Material (PDF) — Figure S1. Six-month changes (%) of clinical variables and plasma concentration of 21 Joslin Kidney Panel (JKP) proteins in individuals treated with glargine and dulaglutide (Dula), stratified by baseline eGFR (A) above or (B) below median of 38 ml/min per 1.73 m2. Table S1. List of 21 proteins associated with the risk of end-stage kidney disease (ESKD) included in the Joslin Kidney Panel (JKP). Table S2. Comparison of baseline plasma concentration (pg/ml) of 21 Joslin Kidney Panel (JKP) proteins between individuals treated with glargine and dulaglutide (Dula). Table S3. Six-month changes (%) for clinical variables and plasma concentration of 21 Joslin Kidney Panel (JKP) proteins in individuals treated with glargine and dulaglutide (Dula), stratified by baseline UACR below or above median (187 mg/g). Table S4. Comparison of 6-month changes in % for clinical variables and plasma concentration of 21 Joslin Kidney Panel (JKP) proteins between individuals treated with glargine and dulaglutide (Dula), stratified by baseline HbA1c below or above median (8.4%). Table S5. Comparison of 6-month changes in % for clinical variables and plasma concentration of 21 Joslin Kidney Panel (JKP) proteins between individuals treated with glargine and dulaglutide (Dula), stratified by baseline eGFR below or above median (32 kg/m2). CONSORT 2025 Checklist. [file mmc1.pdf]

Dulaglutide Effect on Proteins Associated with Chronic Kidney Disease Progression

Brandon E. McFarlin[1a], Sok Cin Tye[2,3a], Eiichiro Satake[2,3], Zaipul I. Md Dom[2,3], Afton Kechter[1], Jonathan M. Wilson[1], Andrzej S. Krolewski[2,3], and Kevin L. Duffin[1*]

Supplementary Material

Supplemental Figure S1. 6-months changes (%) of for clinical variables and plasma concentration of 21 Joslin Kidney Panel (JKP) proteins in individuals treated with insulin glargine (Glargine) and dulaglutide (Dula), stratified by baseline eGFR (A) above or (B) below median 38 ml/min/1.73m^2

Supplementary Table S1. List of 21 proteins associated with the risk of end-stage kidney disease (ESKD) included in the Joslin Kidney Panel (JKP)

Supplementary Table S2. Comparison of baseline plasma concentration (pg/ml) of 21 Joslin Kidney Panel (JKP) proteins between individuals treated with insulin glargine and dulaglutide

Supplementary Table S3. 6-month changes (%) for clinical variables and plasma concentration of 21 Joslin Kidney Panel (JKP) proteins in individuals treated with insulin glargine and dulaglutide, stratified by baseline UACR below or above median (187 mg/g)

Supplementary Table S4. Comparison of 6-month changes in % for clinical variables and plasma concentration of 21 Joslin Kidney Panel (JKP) proteins between individuals treated with insulin glargine and dulaglutide, stratified by baseline HbA1c below or above median (8.4%)

Supplementary Table S5. Comparison of 6-month changes in % for clinical variables and plasma concentration of 21 Joslin Kidney Panel (JKP) proteins between individuals treated with insulin glargine and dulaglutide, stratified by baseline BMI below or above median (32 kg/m2)

Supplementary Table S6. CONSORT 2025 Checklist

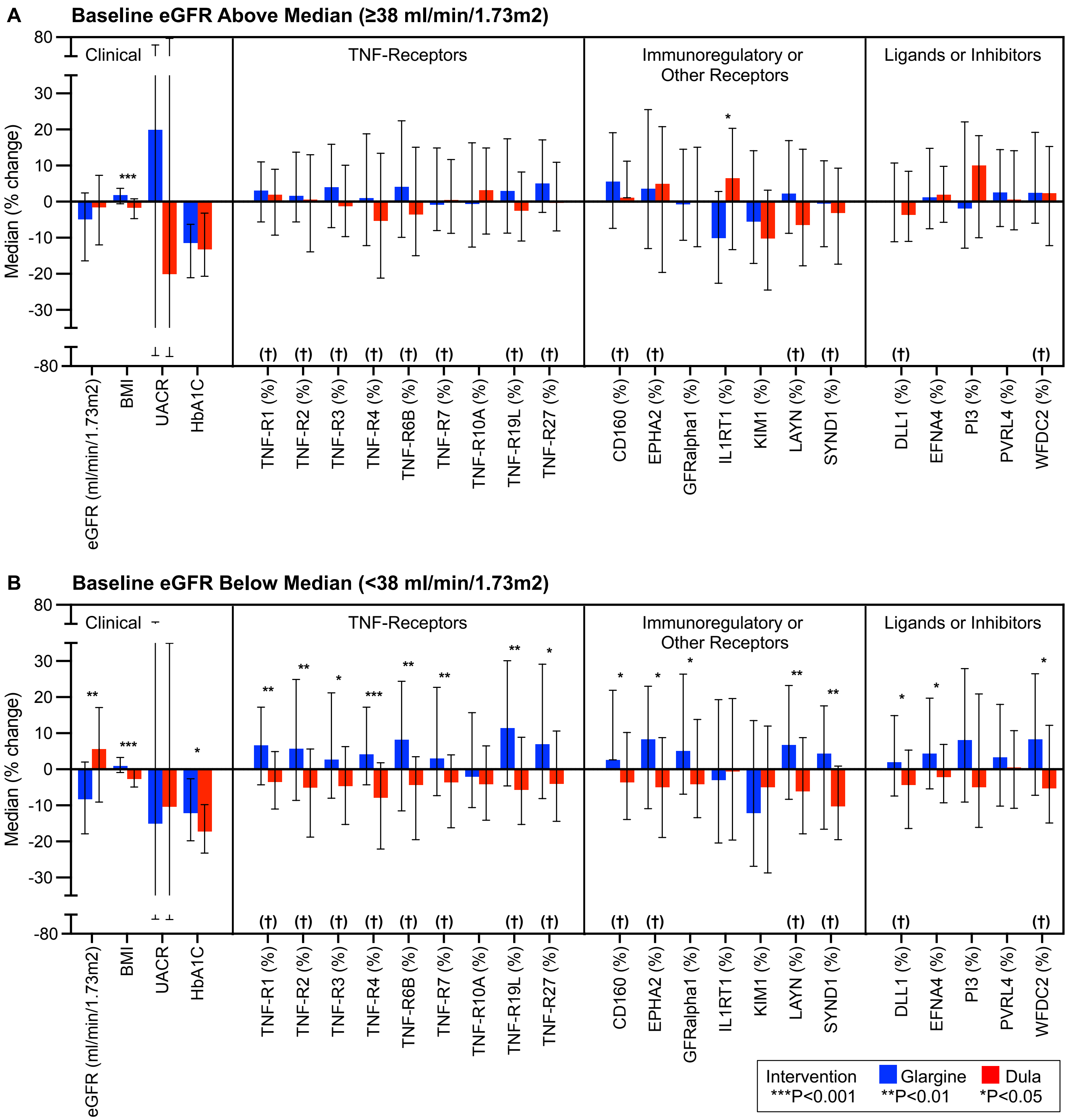

Supplemental Figure S1. 6-months changes (%) of for clinical variables and plasma concentration of 21 Joslin Kidney Panel (JKP) proteins in individuals treated with insulin glargine (Glargine) and dulaglutide (Dula), stratified by baseline eGFR (A) above or (B) below median 38 [illegible]

Supplementary Table S1. List of 21 proteins associated with the risk of end-stage kidney disease (ESKD) included in the Joslin Kidney Panel (JKP)

Protein names on JKP	Protein names on OLINK	Protein Full Name	Entrez Gene Symbol	Intra %CV Average*	Inter %CV Average*
**TNF receptors**					
TNF-R1	TNF-R1	TNF receptor superfamily member 1A	*TNFRSF1A*	4%	12%
TNF-R2	TNF-R2	TNF receptor superfamily member 1B	*TNFRSF1B*	5%	9%
TNF-R3	LTBR	TNF receptor superfamily member 3	*LTBR*	5%	12%
TNF-R4	TNFRSF4	TNF receptor superfamily member 4	*TNFRSF4*	7%	14%
TNF-R6B	TNFRSF6B	TNF receptor superfamily member 6B	*TNFRSF6B*	8%	13%
TNF-R7	CD27	TNF receptor superfamily member 7	*CD27*	5%	11%
TNF-R10A	TNFRSF10A	TNF receptor superfamily member 10A	*TNFRSF10A*	11%	24%
TNF-R19L	RELT	TNF receptor superfamily member 19L	*RELT*	6%	12%
TNF-R27	EDA2R	TNF receptor superfamily member 27	*EDA2R*	7%	11%
**Immunoregulatory or other receptors**					
CD160	CD160	CD160 antigen	*CD160*	6%	10%
EPHA2	EPHA2	Ephrin type-A receptor 2	*EPHA2*	10%	20%
GFRa1	GFR-alpha-1	GDNF family receptor alpha 1	*GFRA1*	7%	17%
IL-1RT1	IL-1RT1	Interleukin-1 receptor type 1	*IL1R1*	6%	16%
KIM1	KIM1	Hepatitis A virus cellular receptor 1	*HAVCR1*	7%	12%
LAYN	LAYN	Layilin	*LAYN*	13%	19%
SYND1	SYND1	Syndecan-1	*SDC1*	7%	14%
**Ligands or inhibitors**					
DLL1	DLL1	Delta-like protein 1	*DLL1*	5%	10%
EFNA4	EFNA4	Ephrin-A4 ligand	*EFNA4*	8%	13%
PI3	PI3	Elafin	*PI3*	4%	11%
PVRL4	PVRL4	Poliovirus receptor-related protein 4-like	*NECTIN4*	6%	12%
WFDC2	WFDC2	WAP four-disulfide core domain protein 2	*WFDC2*	5%	10%

*Intra and inter %CV of Joslin Kidney Panel calculated using four sample controls measured in triplicates from eight experiments.

Supplementary Table S2: Comparison of baseline plasma concentration (pg/ml) of 21 Joslin Kidney Panel (JKP) proteins between individuals treated with insulin glargine and dulaglutide.

Proteins (Aliases)	Insulin Glargine (N=125) Median [IQR] pg/ml	Dulaglutide 1.5mg (N=124) Median [IQR] pg/ml	P Value
**TNF receptors**			
TNF-R1	13,469 [10,975, 18,232]	13,543 [11,275, 16,803]	Ns.
TNF-R2	12,159 [9795, 17162]	12,612 [9,813, 16,316]	Ns.
TNF-R3	199 [151, 284]	192 [163, 249]	Ns.
TNF-R4	14 [10, 19]	14 [11, 17]	Ns.
TNF-R6B	178 [138, 224]	175 [132, 226]	Ns.
TNF-R7	251 [176, 404]	255 [187, 394]	Ns.
TNF-R10A	28.9 [22, 37]	29 [24, 35]	Ns.
TNF-R19L	1,315 [1,013, 1,845]	1,294 [1,062, 1,608]	Ns.
TNF-R27	22 [16, 32]	23 [16, 30]	Ns.
**Immunoregulatory or other receptors**			
CD160	117 [85, 156]	117 [90, 150]	Ns.
EPHA2	32 [23 , 48]	33 [23, 44]	Ns.
GFRa1	1,121 [870, 1,413]	1,045 [835, 1,311]	Ns.
IL1RT1	148 [121, 187]	157 [134, 200]	Ns.
KIM1	441 [286, 658]	461 [290, 846]	Ns.
LAYN	10 [6, 14]	9 [7, 13]	Ns.
SYND1	230 [169, 290]	224 [189, 285]	Ns.
**Ligands or inhibitors**			
DLL1	1,733 [1,409, 2,152]	1,731 [1,416, 2,122]	Ns.
EFNA4	124 [100, 159]	121 [106, 148]	Ns.
PI3	18,896 [14,037, 26,248]	19,370 [14,628, 25,521]	Ns.
PVRL4	150 [115, 205]	152 [121, 203]	Ns.
WFDC2	422 [269, 658]	408 [299, 662]	Ns.

Supplementary Table S3: 6-month changes (in %) for clinical variables and plasma concentration of 21 Joslin Kidney Panel (JKP) proteins in individuals treated with insulin glargine and dulaglutide, stratified by baseline UACR below or above median (187 mg/g)

Biomarkers (6-month changes in %)	Insulin Glargine (N=65)	Dulaglutide (N=60)	**P value	Insulin Glargine (N=60)	Dulaglutide (N=64)	**P value
	UACR < Median	UACR < Median		UACR ≥ Median	UACR ≥ Median	
	6-months change* Median (IQR) %	6-months change* Median (IQR) for %		6-months change* Median (IQR) %	6-months change* Median (IQR) for %	
**Clinical**						
eGFR	-2.6 [-10, 4.7]	2.2 [-4.2, 7.5]	0.061	-11.8 [-20.6, -2.5]	-4.6 [-13.1, 15.2]	0.001
BMI	1.6 [0, 3.7]	-1.7 [-4.8, 0.9]	<0.001	0.8 [-1, 3.3]	-2.7 [-4.9, 0]	<0.001
HbA1c	-10.2 [-18.7, -5.2]	-14.1 [-20.4, -4.7]	0.331	-13.2 [-21.5, -4.6]	-17.6 [-24.1, -8.9]	0.217
UACR	-16.5 [-45.4, 61.9]	5.3 [-46.3, 80.7]	0.784	-17.6 [-54.3, 40.3]	-19.4 [-56.6, 23.3]	0.552
**TNF receptors**						
TNF-R1 (†)	1.8 [-7.1, 11]	0.4 [-8.9, 5.6]	0.190	7.7 [-3.1, 17.6]	-2.8 [-12.3, 6.2]	0.001
TNF-R2 (†)	-0.8 [-7.6, 13.6]	0.3 [-16.3, 8.4]	0.188	8.1 [-7.2, 23]	-4.6 [-14.9, 8]	0.004
TNF-R3 (†)	0.1 [-9.5, 15.3]	-1.8 [-9.4, 14.3]	0.609	6 [-5.6, 20.7]	-4 [-15.6, 5.3]	0.002
TNF-R4 (†)	1 [-12.5, 16.8]	-3.2 [-19.6, 11.5]	0.131	4 [-5.3, 21.3]	-7.9 [-25.6, 1.8]	<0.001
TNF-R6B (†)	4.6 [-9.9, 22.4]	-3.3 [-15.6, 12.3]	0.041	6.1 [-13, 26.1]	-5.2 [-18.9, 5.2]	0.007
TNF-R7 (†)	-1.4 [-8, 11.3]	0.2 [-10.5, 8]	0.888	5 [-7.3, 23]	-3.6 [-14.3, 9.2]	0.009
TNF-R10A	-0.7 [-11.3, 16.3]	3.4 [-7.5, 14.9]	0.542	-2 [-11.5, 15.8]	-4.8 [-14.1, 7.4]	0.151
TNF-R19L (†)	0.8 [-9.8, 15.2]	-1.3 [-11.5, 11.5]	0.419	14 [-4, 30.1]	-6 [-15.1, 7.9]	<0.001
TNF-R27 (†)	2.6 [-7.7, 16.1]	-1.6 [-10.9, 11.8]	0.210	7.2 [-1.7, 33.7]	-2.8 [-11.2, 9.8]	0.003
**Immunoregulatory or other receptors**						
CD160 (†)	1 [-8.5, 16.3]	-1.8 [-9.7, 10.5]	0.305	6.1 [-5.5, 22.6]	1.3 [-15.4, 10.3]	0.017
EPHA2 (†)	1.9 [-12.9, 20.1]	3.8 [-16.6, 20.8]	0.606	10.5 [-11.8, 26]	-7.3 [-21.3, 10.2]	0.005
GFRa1	-1.9 [-10.7, 14.5]	2.3 [-11.6, 17.8]	0.911	6.1 [-4.4, 25.6]	-5.5 [-14.4, 10]	0.008
IL1RT1	-9.1 [-20.9, 5]	7.4 [-12.2, 28.9]	0.014	-4.3 [-23.1, 16]	-4.9 [-17.9, 15.9]	0.766
KIM1	-5.9 [-19.5, 14.1]	-3.7 [-23.4, 20.7]	0.947	-10.6 [-28.8, 10.3]	-10.9 [-28, 1.6]	0.458
LAYN (†)	-0.5 [-10.3, 15.2]	-1.8 [-18.2, 14.1]	0.312	10.9 [-5.6, 26.8]	-10.2 [-17.7, 8.8]	<0.001
SYND1 (†)	-1.3 [-14.9, 11.3]	-1.8 [-17.2, 12.5]	0.487	2.4 [-10.9, 19.1]	-10.5 [-23, 0]	<0.001
**Ligands or inhibitors**						
DLL1 (†)	-2.9 [-11.1, 10.8]	-0.3 [-8.9, 10.8]	0.911	3.7 [-6.6, 15.2]	-8.3 [-16.6, 3.3]	0.001
EFNA4	-0.6 [-7.5, 13.9]	4.6 [-4.6, 10.8]	0.472	5.6 [-4.6, 24.5]	-3.8 [-10.7, 4.6]	0.002
PI3	-1.9 [-13.2, 18.8]	13.1 [-8, 22.1]	0.105	9.2 [-7.9, 29.1]	-6.3 [-13.9, 13.2]	0.011

PVRL4	-0.7 [-9.8, 12.6]	3 [-7.9, 14.3]	0.644	5.2 [-8.7, 23]	-0.8 [-10.3, 10]	0.066
WFDC2 (†)	0.9 [-7.5, 15.6]	1.3 [-12.8, 13.2]	0.575	17.6 [-5.1, 29.8]	-3.6 [-14.9, 13.7]	0.001

* 6-month change in % was computed by formula [(6 month measurement – baseline/ baseline) x 100%].

** P-values represent the results of the Mann-Whitney U test comparing insulin glargine versus dulaglutide.

(†) Denotes 14 key JKP proteins, see legend to Table 2.

Supplementary Table S4. Comparison of 6-month changes in % for clinical variables and plasma concentration of 21 Joslin Kidney Panel (JKP) proteins between individuals treated with insulin glargine and dulaglutide, stratified by baseline HbA1c below or above median (8.4%)

Biomarker (%) (6-month change)	Insulin Glargine (N=68)	Dulaglutide (N=51)	[1]P value	Insulin Glargine (N=57)	Dulaglutide (N=73)	[2]P value
	HbA1c < Median	HbA1c < Median		HbA1c ≥ Median	HbA1c ≥ Median	
	6-months change* Median (IQR) %	6-months change* Median (IQR) %		6-months change* Median (IQR) %	6-months change* Median (IQR) %	
**Clinical**						
eGFR	-5.2 [-15.6, 3.4]	0.5 [-10.1, 12.3]	0.081	-6.9 [-20.9, 0]	1.7 [-10.5, 11.6]	0.001
BMI	1 [-1.3, 3.1]	-3.7 [-5.4, 0.2]	<0.001	1.7 [0, 3.9]	-1.7 [-4.3, 0.2]	<0.001
HbA1c	-8.8 [-14.1, -0.9]	-10.1 [-18.1, -4]	0.133	-18.4 [-25, -9.6]	-18.4 [-23.8, -10.6]	0.638
UACR	-10.3 [-39.5, 65.6]	-8.3 [-51.6, 33.1]	0.533	-26.2 [-59.7, 34.9]	-18.8 [-56.3, 48.4]	0.633
**TNF receptors**						
TNF-R1 (†)	4.8 [-7, 15.4]	0.3 [-8.6, 5.6]	0.121	5.6 [-3.2, 13]	-2.8 [-12.1, 6]	0.005
TNF-R2 (†)	2 [-6.3, 19.6]	-2.8 [-14.8, 9.1]	0.043	6.2 [-8.1, 20.6]	-2 [-15.9, 7.1]	0.031
TNF-R3 (†)	0.1 [-8, 20.9]	-4.3 [-10.2, 16.5]	0.243	4.8 [-6.5, 15.3]	-2.1 [-14.1, 7.5]	0.010
TNF-R4 (†)	0.9 [-10.9, 16.7]	-5.2 [-17.7, 9.6]	0.071	4.7 [-6.8, 24]	-8.1 [-25.3, 5.6]	0.001
TNF-R6B (†)	5.6 [-7.5, 24.5]	-4.9 [-21.6, 7.2]	0.002	2.1 [-15.7, 23.3]	-3.4 [-15.1, 10.9]	0.084
TNF-R7 (†)	1.4 [-7.3, 18.9]	0.5 [-9.2, 10.8]	0.553	2.5 [-10.4, 19]	-3.6 [-13.7, 7.3]	0.062
TNF-R10A	-1.4 [-10.4, 18.7]	1.3 [-9.1, 14.9]	0.998	-1.8 [-13.7, 13.9]	-2.2 [-13.8, 7.8]	0.442
TNF-R19L (†)	2.2 [-10.6, 20.8]	-4 [-12.4, 8.9]	0.085	6.2 [-4, 22.7]	-5.9 [-13.4, 7.7]	0.002
TNF-R27 (†)	2.6 [-8.2, 20.8]	-1.1 [-10.9, 12.7]	0.322	8.6 [-2, 27]	-3.4 [-12.3, 10.3]	0.001
**Immunoregulatory or other receptors**						
CD160 (†)	1.3 [-7.5, 16.2]	-0.2 [-9.9, 10.3]	0.284	6.6 [-8.5, 22.2]	1.9 [-13.1, 10.6]	0.017
EPHA2 (†)	3.5 [-13, 24.7]	-0.4 [-18, 22.9]	0.494	9 [-8.1, 25.5]	-6.2 [-19.1, 12.7]	0.008
GFRa1	2.8 [-10.2, 14.6]	2.4 [-11, 18]	1.000	5.1 [-10.1, 26.2]	-4.8 [-15.1, 10.4]	0.022
IL1RT1	-1 [-15.8, 18.3]	7.2 [-8.7, 17.7]	0.230	-12.3 [-27, 5]	-5.3 [-18.2, 19.8]	0.033
KIM1	0.4 [-14.1, 24.5]	-5.1 [-23.5, 14.8]	0.067	-17.1 [-35, 4.1]	-9.8 [-27.6, 5.5]	0.221
LAYN (†)	1.2 [-7.5, 23.5]	-2 [-14.8, 15.3]	0.172	10 [-10.6, 18.5]	-10.2 [-19, 10.2]	0.001
SYND1 (†)	-0.5 [-16.6, 10.9]	-4.7 [-17.3, 5.8]	0.132	1.2 [-10.2, 18.9]	-7.1 [-20.9, 3.6]	0.003
**Ligands or inhibitors**						
DLL1 (†)	0.1 [-9, 14.9]	-1.3 [-11.1, 6.6]	0.160	1.3 [-9, 11.2]	-6.1 [-14.7, 7.4]	0.067
EFNA4	0.7 [-6.7, 16.8]	5.2 [-5.1, 11.2]	0.866	3.8 [-4.2, 16.8]	-2.2 [-9.1, 4.7]	0.018
PI3	2.5 [-11.7, 22.3]	6.8 [-7.2, 20.6]	0.693	1 [-12.9, 24]	-7.3 [-12.2, 19]	0.192

PVRL4	-0.9 [-9.9, 18]	1.4 [-7.9, 13.6]	0.878	3.5 [-7.4, 16.4]	-0.2 [-10.3, 10.6]	0.194
WFDC2 (†)	3.7 [-7.1, 23.2]	1.8 [-12.9, 15.3]	0.236	11 [-5.2, 26.4]	-3.9 [-14.6, 12.1]	0.007

* 6-month change in % was computed by formula [(6 month measurement – baseline/ baseline) x 100%].

** P-values represent the results of the Mann-Whitney U test comparing insulin glargine versus dulaglutide.

(†) Denotes 14 key JKP proteins, see Legend to Table 2.

Supplementary Table S5. Comparison of 6-month changes in % for clinical variables and plasma concentration of 21 Joslin Kidney Panel (JKP) proteins between individuals treated with insulin glargine and dulaglutide, stratified by baseline BMI below or above median (32 kg/m^2)

Biomarkers (%) (6-month changes)	Insulin Glargine (N=59)	Dulaglutide (N=66)	**P value	Insulin Glargine (N=66)	Dulaglutide (N=58)	**2P value
	BMI < Median	BMI < Median		BMI ≥ Median	BMI ≥ Median	
	6-months change* Median (IQR) %	6-months change* Median (IQR) %		6-months change* Median (IQR) %	6-months change* Median (IQR) %	
**Clinical**						
eGFR	-8.2 [-15.6, 0]	2.4 [-9.9, 12.3]	0.001	-3.2 [-18.6, 3.2]	0.5 [-12.8, 11.4]	0.064
BMI	1.4 [-0.6, 3.8]	-1.6 [-4.1, 1.2]	<0.001	1.1 [-0.8, 3.4]	-2.6 [-5.5, -0.4]	<0.001
HbA1c	-12.1 [-21.3, -2.3]	-16.9 [-22.5, -9.8]	0.060	-11.2 [-19.3, -5.8]	-13.2 [-20.6, -3.4]	0.930
UACR	-31.8 [-64.1, 32.3]	-10.4 [-47.3, 46.1]	0.180	-1.7 [-37.1, 84.5]	-23.8 [-56.2, 31.4]	0.129
**TNF receptors**						
TNF-R1 (†)	5.5 [-7.6, 13.6]	0.8 [-6.6, 11.3]	0.535	5.4 [-2, 14.6]	-3.6 [-12.7, 3.7]	<0.001
TNF-R2 (†)	2.3 [-6.8, 21.3]	1.2 [-10.5, 9.2]	0.293	4.9 [-7.8, 14.2]	-5.9 [-18.3, 5.1]	0.001
TNF-R3 (†)	4 [-8.5, 17.9]	0.7 [-10.9, 16.3]	0.463	2 [-6.1, 16.9]	-7.2 [-15.5, 3.2]	0.001
TNF-R4 (†)	4.2 [-9.9, 25.6]	-3.6 [-19.1, 11.7]	0.053	2.4 [-9.7, 17.1]	-9 [-21.6, 1.9]	<0.001
TNF-R6B (†)	2.5 [-11, 25.6]	-3.3 [-15.6, 11.8]	0.036	7.2 [-11.7, 23.6]	-5.3 [-19.4, 9.1]	0.003
TNF-R7 (†)	-0.9 [-6.9, 19.6]	-0.7 [-11.4, 10.2]	0.512	2.6 [-7.9, 17.2]	-4.9 [-11.7, 3.8]	0.018
TNF-R10A	-2.9 [-13, 17.3]	3.4 [-10.4, 13]	0.548	2 [-10.3, 15]	-5.1 [-13.6, 5.4]	0.077
TNF-R19L (†)	3.2 [-7.5, 20.3]	1.1 [-11.4, 14.2]	0.232	5 [-7.4, 26.6]	-7.2 [-14.8, 4.4]	<0.001
TNF-R27 (†)	5 [-6.4, 22.1]	-1.1 [-10.4, 13.7]	0.240	6.3 [-5, 23.8]	-4 [-12.5, 8.4]	0.002
**Immunoregulatory or other receptors**						
CD160 (†)	5.2 [-8.7, 19.2]	3.6 [-9.9, 12.1]	0.493	1.3 [-5.5, 19.4]	-3.6 [-13.9, 7.4]	0.004
EPHA2 (†)	2.1 [-14.9, 32.9]	4.7 [-14.6, 23.9]	0.837	8.4 [-7.7, 19]	-7.2 [-23.9, 7.2]	0.001
GFRa1	2.9 [-11.5, 21.3]	4.3 [-10, 21.2]	0.761	3.5 [-7.9, 22.6]	-7.3 [-14.5, 6]	0.002
IL1RT1	-10.3 [-23, 3.6]	4.3 [-10, 19.8]	0.021	-3.9 [-16.6, 12.9]	-2.1 [-21.6, 18.3]	0.669
KIM1	-5.9 [-20.8, 16.7]	-9.6 [-23.5, 13.8]	0.445	-10.3 [-21.5, 10.3]	-9.2 [-27.3, 5]	0.705
LAYN (†)	3.3 [-9.5, 21.2]	1.1 [-13.9, 13.7]	0.214	2.9 [-8, 20]	-13.1 [-19.1, 6.4]	<0.001
SYND1 (†)	-0.5 [-14.8, 20.3]	-2.4 [-15.1, 6.9]	0.183	0.2 [-12.9, 13.6]	-11.8 [-23.5, 1.2]	0.001
**Ligands or inhibitors**						
DLL1 (†)	0.2 [-12.5, 12]	0.3 [-9.8, 8.9]	0.884	1.6 [-6.9, 14.5]	-8.5 [-16.3, 1.3]	<0.001
EFNA4	1.2 [-9.9, 16.2]	1.6 [-6.7, 11.3]	0.880	3.7 [-4.4, 16.8]	-1.6 [-11.9, 5.4]	0.005
PI3	1 [-10, 23.3]	8.6 [-11.2, 21.6]	0.919	2.5 [-12.6, 22.9]	-3.9 [-13, 13.7]	0.201

PVRL4	3.1 [-9.3, 14.5]	3.9 [-7.9, 14.7]	0.757	2.1 [-8.3, 18]	-2.9 [-10.4, 7.9]	0.084
WFDC2 (†)	2.7 [-6.6, 23.3]	-0.8 [-14.2, 13.1]	0.085	11.7 [-6.2, 25]	-2.7 [-12.6, 13.6]	0.018

* 6-month change in % was computed by formula [(6 month measurement – baseline/ baseline) x 100%].

** P-values represent the results of the Mann-Whitney U test comparing glargine versus Dula.

(†) Denotes 14 key JKP proteins, see legend to Table 2.

Supplementary Table S6. CONSORT 2025 Checklist

Section/topic	No	CONSORT 2025 checklist item description	Reported on page no.
**Title and abstract**			
Title and structured abstract	1a	Identification as a randomised trial	n/a
	1b	Structured summary of the trial design, methods, results, and conclusions	2-3
**Open science**			
Trial registration	2	Name of trial registry, identifying number (with URL) and date of registration	5
Protocol and statistical analysis plan	3	Where the trial protocol and statistical analysis plan can be accessed	5
Data sharing	4	Where and how the individual de-identified participant data (including data dictionary), statistical code and any other materials can be accessed	16
Funding and conflicts of interest	5a	Sources of funding and other support (eg, supply of drugs), and role of funders in the design, conduct, analysis and reporting of the trial	16-17
	5b	Financial and other conflicts of interest of the manuscript authors	16-17
**Introduction**			
Background and rationale	6	Scientific background and rationale	3-5
Objectives	7	Specific objectives related to benefits and harms	3-5
**Methods**			
Patient and public involvement	8	Details of patient or public involvement in the design, conduct and reporting of the trial	n/a

Trial design	9	Description of trial design including type of trial (eg, parallel group, crossover), allocation ratio, and framework (eg, superiority, equivalence, non-inferiority, exploratory)	5
Changes to trial protocol	10	Important changes to the trial after it commenced including any outcomes or analyses that were not prespecified, with reason	n/a
Trial setting	11	Settings (eg, community, hospital) and locations (eg, countries, sites) where the trial was conducted	n/a
Eligibility criteria	12a	Eligibility criteria for participants	5
	12b	If applicable, eligibility criteria for sites and for individuals delivering the interventions (eg, surgeons, physiotherapists)	n/a
Intervention and comparator	13	Intervention and comparator with sufficient details to allow replication. If relevant, where additional materials describing the intervention and comparator (eg, intervention manual) can be accessed	5
Outcomes	14	Prespecified primary and secondary outcomes, including the specific measurement variable (eg, systolic blood pressure), analysis metric (eg, change from baseline, final value, time to event), method of aggregation (eg, median, proportion), and time point for each outcome	6-7
Harms	15	How harms were defined and assessed (eg, systematically, non-systematically)	n/a
Sample size	16a	How sample size was determined, including all assumptions supporting the sample size calculation	n/a
	16b	Explanation of any interim analyses and stopping guidelines	n/a
Randomisation:			
Sequence generation	17a	Who generated the random allocation sequence and the method used	n/a
	17b	Type of randomisation and details of any restriction (eg, stratification, blocking and block size)	n/a

			Reported on page no.
Allocation concealment mechanism	18	Mechanism used to implement the random allocation sequence (eg, central computer/telephone; sequentially numbered, opaque, sealed containers), describing any steps to conceal the sequence until interventions were assigned	n/a
Implementation	19	Whether the personnel who enrolled and those who assigned participants to the interventions had access to the random allocation sequence	n/a
Blinding	20a	Who was blinded after assignment to interventions (eg, participants, care providers, outcome assessors, data analysts)	n/a
	20b	If blinded, how blinding was achieved and description of the similarity of interventions	n/a
Statistical methods	21a	Statistical methods used to compare groups for primary and secondary outcomes, including harms	6-7
	21b	Definition of who is included in each analysis (eg, all randomised participants), and in which group	5
	21c	How missing data were handled in the analysis	5
	21d	Methods for any additional analyses (eg, subgroup and sensitivity analyses), distinguishing prespecified from post hoc	n/a
**Results**			
Participant flow, including flow diagram	22a	For each group, the numbers of participants who were randomly assigned, received intended intervention, and were analysed for the primary outcome	n/a or 5
	22b	For each group, losses and exclusions after randomisation, together with reasons	n/a or 5
Recruitment	23a	Dates defining the periods of recruitment and follow-up for outcomes of benefits and harms	n/a
	23b	If relevant, why the trial ended or was stopped	n/a

Intervention and comparator delivery	24a	Intervention and comparator as they were actually administered (eg, where appropriate, who delivered the intervention/comparator, how participants adhered, whether they were delivered as intended (fidelity))	n/a
	24b	Concomitant care received during the trial for each group	n/a
Baseline data	25	A table showing baseline demographic and clinical characteristics for each group	Table 1
Numbers analysed, outcomes and estimation	26	For each primary and secondary outcome, by group:<br>● the number of participants included in the analysis<br>● the number of participants with available data at the outcome time point<br>● result for each group, and the estimated effect size and its precision (such as 95% confidence interval)<br>● for binary outcomes, presentation of both absolute and relative effect size	n/a
Harms	27	All harms or unintended events in each group	n/a
Ancillary analyses	28	Any other analyses performed, including subgroup and sensitivity analyses, distinguishing pre-specified from post hoc	n/a
**Discussion**			n/a
Interpretation	29	Interpretation consistent with results, balancing benefits and harms, and considering other relevant evidence	11-15
Limitations	30	Trial limitations, addressing sources of potential bias, imprecision, generalisability, and, if relevant, multiplicity of analyses	15

Citation: Hopewell S, Chan AW, Collins GS, Hróbjartsson A, Moher D, Schulz KF, et al. CONSORT 2025 Statement: updated guideline for reporting randomised trials. BMJ. 2025; 388:e081123. https://dx.doi.org/10.1136/bmj-2024-081123
© 2025 Hopewell et al. This is an Open Access article distributed under the terms of the Creative Commons Attribution License (https://creativecommons.org/licenses/by/4.0/), which permits unrestricted use, distribution, and reproduction in any medium, provided the original work is properly cited.

*We strongly recommend reading this statement in conjunction with the CONSORT 2025 Explanation and Elaboration and/or the CONSORT 2025 Expanded Checklist for important clarifications on all the items. We also recommend reading relevant CONSORT extensions. See www.consort-spirit.org.
